# Gender and Internal Geographical Mobility in Europe: A Comparative Analysis of Family and Employment Over the Life Course

**DOI:** 10.1007/s10680-025-09763-5

**Published:** 2025-12-23

**Authors:** Hanne Gaukel, Roberto Impicciatore, Nazareno Panichella, Antonina Zhelenkova

**Affiliations:** 1 Department of Social and Political Sciences, Università degli Studi di Milano, Milan, Italy; 2 Department of Statistical Sciences ‘Paolo Fortunati’, Alma Mater Studiorum - Università di Bologna, Bologna, Italy

**Keywords:** internal migration, geographical mobility, gender, employment, partnership, parenthood, Europe

## Abstract

This article presents an investigation into the gendered outcomes of inter-regional moves in six European countries, adopting a life-course perspective. Analyses are based on retrospective data from SHARELIFE for birth cohorts from the 1930s to 1960s in France, Germany, Italy, Poland, Spain, and Sweden. Linear probability panel models with fixed effects are used to examine the association between inter-regional migration and employment status over time, while assessing whether it differs by gender and family status. Results show that men experience improved employment probabilities following migration, regardless of their family status, and that these outcomes are consistent across countries. Moreover, the likelihood of employment for men continues to gradually increase several years after the move. In contrast, inter-regional mobility favours single women more than partnered women, especially mothers. Results, however, do not confirm a pattern of continued disadvantages, as mobility does not further reduce the employment prospects of mothers over time. The largest differences in the association between geographical mobility and employment between single women and mothers are observed in Mediterranean countries, while in more egalitarian countries like Sweden these differences are comparatively small.

## Introduction

Inter-regional moves are a form of geographical mobility that takes place within national borders, involving relocation from one region of a country to another. Such moves are expected to improve occupational opportunities, with economists, sociologists, and demographers having long recognised the potential benefits of mobility on labour market outcomes (Bernard, 2023; Blau & Duncan, 1967; Mulder & van Ham, 2005). However, empirical evidence suggests that these positive outcomes are not evenly distributed between men and women. Women, in particular, often face additional social and economic penalties in the mobility process, especially when they are married or have children (Cooke, 2003). In contrast, men experience consistently positive returns, regardless of marital status and family concerns.

To explain these disparities, theories on gender and family mobility have emerged, highlighting uneven decision-making processes within couples. Typically, men’s occupational status and earning potential are prioritised in mobility-related decisions, even in cases when women’s prospects of occupational attainment are higher (Nisic & Melzer, 2016). Therefore, family status and institutionalised gender roles must be considered as crucial factors in the mobility process, because moving alone or with a partner, and potentially with children, may affect employment opportunities for men and women in distinct ways (Panichella & Cantalini, 2023). The aim of this study is to examine the role of gender, partnership, and parenthood status in shaping the relationship between inter-regional mobility and employment status in a cross-national framework over time.

To pursue these objectives, we apply linear probability panel models with fixed effects to retrospective data from the SHARELIFE survey, which provides comparative information on the life courses of individuals interviewed at age 50 and over, including birth cohorts from the 1930s to the 1960s. We focus on six European countries: France, Germany, Italy, Poland, Spain, and Sweden.

We thus provide two main contributions to the literature. First, we examine how the association between inter-regional mobility and employment over time differs by gender and family status. This temporal and life-course-oriented approach enables us to assess whether mobility exerts an immediate, delayed, or lasting influence on employment. Second, by comparing six European countries that differ in levels of social inequality (Breen & Müller, 2020), labour market structure and regulation (Barbieri & Cutuli, 2016; Esping-Andersen & Regini, 2000), gender roles (Pfau-Effinger, 1998, 2012), gender inequality in the labour market (Orloff, 1993; Stier et al., 2001), and welfare systems (Esping-Andersen, 1990), we extend existing knowledge by situating cross-national variation in employment–mobility patterns within the broader institutional and cultural contexts that characterise these settings.

## Background

### Inter-regional Mobility and Gendered Employment Outcomes

Geographical mobility is often conceptualised in neo-classical economics as an investment in occupational advancement: despite the initial costs of moving, the long-term gains justify the price (Greenwood, 1975; Sjaastad, 1962). It is an investment with benefits, risks, and costs, resulting in a (self-)selective process, in which individuals with certain personal characteristics, such as higher levels of education or more advantaged social backgrounds, are more likely to move to find better opportunities and obtain more favourable outcomes (Panichella & Cantalini, 2023; Bernard & Bell, 2018).

The association between geographical mobility and employment outcomes unfolds within broader life contexts shaped by gender, family, and socio-demographic circumstances (Brandén & Haandrikman, 2019; Impicciatore & Panichella, 2019; Vidal & Lersch, 2019). Men and women do not benefit equally from geographical mobility: men tend to experience more consistently positive occupational outcomes from moving, regardless of their individual characteristics, while for women the effects are often unclear or negative (Boyle et al., 2009; Cooke et al., 2009; De Jong & Graefe, 2008). Gender differences in the impact of geographical mobility on employment outcomes have been highly linked to marital status (Mulder & Wagner, 1993), with marriage affecting men’s and women’s occupational outcomes differently (Panichella & Cantalini, 2023). Geographical mobility can depress a woman’s earnings, result in her exit from employment, and substantially reinforce traditional gender roles within a couple (Boyle et al., 2009; Cooke et al., 2009; De Jong & Graefe, 2008; Sandell, 1977). Explanations for differences in occupational outcomes and employment status can then often be drawn back to gender roles and family (Cooke, 2008; Kulu & Milewski, 2007).

Two main arguments attempt to explain this phenomenon (Panichella & Impicciatore, 2024). According to the human capital theories of family migration, couples move when they expect a net positive gain for the couple as a whole, even if one partner experiences a personal net loss and becomes a ‘tied mover’ (Mincer, 1978). Because such theories are formulated as gender-neutral, they do not incorporate social norms or intra-household power dynamics, and therefore cannot account for the fact that women may remain tied movers even when they have a higher earning potential, as their employment prospects often receive less weight in family decision-making (Cooke, 2008; Duncan & Perrucci, 1976).

A second argument is offered by gender-asymmetric theories, which posit that migration decisions are shaped not only by economic considerations but also by gendered beliefs and role expectations (Bielby & Bielby, 1992). Under this framework, even if a woman has a higher earning potential than her husband, this may be discounted if the husband is the main breadwinner. So, couples and families are more likely to move for a male partner’s career than a female’s (Cooke, 2003; Smits et al., 2003). Thus, given the traditional gender roles that exist in male-dominant breadwinner societies, geographical mobility is generally a way to promote career advancement for men, whereas for women, it is a response to their partners’ movements rather than the pursuit of their own occupational opportunities (Taylor, 2007).

Children must also be considered regarding geographical mobility and participation in the labour market. Notably, women tend to have less bargaining power within the family when they are parents, due to more vulnerable labour market positions arising from family obligations (Neyer et al., 2013), a situation which does not occur to the same extent for men. The relationship between geographical mobility and family, such as partnership and children is complex (Mulder & Wagner, 1993; Vidal & Huinink, 2019), requiring increased attention to these factors over time, and their potential interplay with employment.

Following previous literature, we expect that inter-regional mobility benefits men’s employment more than women’s (*Hypothesis 1*), reflecting gendered differences in the returns to mobility. Furthermore, in line with human capital theory and gender-asymmetry theory, we expect that women with family obligations – i.e., partnered women and, in particular, mothers – experience less favourable employment outcomes compared to single women (*Hypothesis 2*), due to the gendered dynamics of decision-making, family commitments, and labour market prioritisation within couples.

### The Temporal Patterns of Geographical Mobility and Employment

Although previous studies have attempted to address the relationship between geographical mobility and occupational attainment, the temporal patterns of these associations remains insufficiently understood. Timing effects on employment can manifest in several ways: geographical mobility may have immediate effects (e.g., job loss or rapid reemployment), delayed effects (e.g., gradual integration into the labour market), or even anticipatory effects (e.g., labour market disengagement prior to migration) (Panichella, 2025). These dynamics may vary according to gender, family composition, and life stage. Indeed, the timing of mobility may contribute to processes of cumulative (dis)advantage over time, whereby an individual’s personal conditions at the time of geographical mobility can shape employment trajectories that persist over the life course (DiPrete & Eirich, 2006). For example, when married women become secondary earners and primary caregivers in the family, this dynamic can limit opportunities for advancement, reduce earnings, and reinforce unequal distributions of paid work and family responsibilities. For men, by contrast, mobility-related improvements in occupational attainment could accumulate, contributing to long-term advantages and widening gender inequalities over time.

Research consistently documents that geographical mobility is associated with changes in women’s employment and earnings trajectories. Clark and Withers (2002) found that geographical mobility temporarily reduced married women’s income levels, although these effects diminished within a year as employment levels recovered. Conversely, longitudinal evidence indicates that geographical mobility can have lasting effects on women’s earnings, with some studies showing that post-move recovery takes several years (Cooke et al., 2009). More recent research highlights that such effects are shaped by broader social and societal factors. For instance, Panichella (2025) finds that in Italy, men tend to show immediate occupational improvements after an internal move, whereas women’s labour market integration progresses more gradually.

Overall, the evidence suggests considerable heterogeneity in women’s post-move employment trajectories, with patterns ranging from short-term adjustments to more persistent changes. We posit that women, especially those who are partnered and mothers, display less sustained improvements in employment following geographical mobility (*Hypothesis 3a*). We expect that men, instead, show more stable and positive employment patterns over time following geographical mobility (*Hypothesis 3b*).

### Cross-National Variation in Gendered Mobility Effects in Europe

At the cross-national level, the literature does not show consistent results as to gendered mobility effects in Europe, as most existing studies are based on single-country analyses that have produced diverse findings. A substantial part of this literature draws on studies from the United States (Cooke, 2001, 2003) and Great Britain (Boyle et al., 1999, 2001, 2003; Cooke et al., 2009) and documents persistent disadvantages for women in terms of labour market outcomes, consistent with human capital models of family migration.

However, these studies are based on countries that share several institutional characteristics, as liberal welfare states with similar gender norms and broadly comparable labour market regulations. By contrast, studies focusing on other national contexts have identified patterns that differ in various ways. Evidence shows that partnered women appear to experience more favourable post-move employment patterns in West Germany than in East Germany, despite the West’s more traditional gender norms (Nisic & Melzer, 2016). In other words, despite their more egalitarian attitudes and socialization in a socialist context – where female employment was often encouraged and institutionally supported (Matysiak, 2009) – couples from East Germany tend to adopt a more traditional orientation toward gender roles when making migration decisions (Melzer, 2013). Such findings underscore that the gendered labour market outcomes of mobility cannot be disentangled from the macro-level policy and cultural environments in which they occur, particularly from the degree of institutional support for maternal employment (Esping-Andersen, 1990).

Similar to West Germany, several other European countries show smaller gender differences than those typically observed in the United States and Great Britain. In France, long-distance moves are associated with short-term reductions in women’s employment, although those who remain employed do not appear to experience pay losses after moving (Pailhé & Solaz, 2008). In the Netherlands, Mulder and van Ham (2005) found that, unlike men, who consistently experience a positive effect of geographical mobility on occupational achievement, women tend to face negative effects immediately after the move, although these short-term disadvantages generally fade within a few years.

Evidence from Southern Europe adds further heterogeneity to the interplay between gender, mobility, and labour market outcomes. In Italy, characterised by strong family ties, weak state family policies, and relatively low levels of female employment, evidence indicates that married women are penalised relative to men, likely due to traditional family structures and limited reconciliation policies (Adsera, 2005; Panichella & Cantalini, 2023). However, in the Southern European context more broadly, evidence remains sparse and at times contradictory (Reher, 1998). For instance, in Spain, geographical mobility appears to benefit women’s occupational attainment (Mulder et al., 2022). This limited and inconsistent evidence across Southern European countries highlights the need for additional research in this area.

Taken together, these patterns suggest that the gendered consequences of geographical mobility may be shaped by welfare state regimes. We do not expect strong cross-country differences for men, as men’s labour market trajectories tend to be less sensitive to family responsibilities and institutional constraints. For women, however, the association between mobility and employment tends to be conditioned by family status across countries with distinct institutional configurations. A reduction in employment associated with the geographical mobility of partnered women and mothers may be mitigated in more supportive contexts, particularly social-democratic welfare states, marked by extensive public childcare provisions, strong support for maternal employment, and policies promoting dual-earner or dual-career arrangements (Esping-Andersen, 1990, 1999), and, to a lesser extent, conservative–corporatist regimes, where earnings-related benefits and family-oriented policies nonetheless provide moderate support for working mothers (Matysiak & Vignoli, 2008). Conversely, differences in the employment chances of partnered women and mothers versus single women are expected to be more pronounced in Mediterranean welfare regimes, marked by limited public childcare provisions and low female employment rates, which reinforce traditional gender divisions of labour (Adsera, 2005; Boeri et al., 2005; Ferrera, 1996; Saraceno & Keck, 2010), and in Eastern European regimes, which combine legacies of high female employment with limited work-family reconciliation policies, especially after the fall of socialism (Matysiak, 2011; Szelewa & Polakowski, 2008).

Thus, our final hypotheses are as follows: the employment penalty associated with geographical mobility for partnered women and mothers, compared to single women, is expected to be larger in countries characterized by Mediterranean and Eastern European welfare regimes than in the countries with social-democratic and conservative–corporatist welfare regimes (*Hypothesis 4a*). By contrast, we do not expect the broader institutional context to significantly affect men’s employment outcomes (*Hypothesis 4b*).

## Data and Methods

### Data

Data used in this study comes from the Survey of Health, Ageing and Retirement in Europe (SHARE), a collection of nationally representative longitudinal surveys of the population aged fifty and over residing in Europe. Waves 3 and 7 recall life histories, conducted in 2008–09 and 2017, respectively, and are known as SHARELIFE. These are retrospective life history questionnaires on geographical mobility, education, occupational history, and family and reproductive behaviour (SHARE-ERIC, 2024a, 2024b). Despite the retrospective nature of the data, it has been shown that respondents can accurately recall events in the distant past, especially when supported by modern survey techniques like life grids (Berney & Blane, 2003; Havari & Mazzonna, 2015).

We merged data from both waves to create a harmonised dataset, excluding observations with inconsistencies and lacking information on the region of residence for all residential episodes. Analysis is restricted to individuals born between 1930 and 1969, so analyses refer primarily to outcomes observed in the second half of the twentieth century. The selected observation window starts at age 15 and ends at age 40, a period when individuals are most likely to be active in processes of family formation, geographical mobility, and changes in employment status. The final sample includes 25,353 individuals who are observed over the life course, of whom 6,295 (24.8%) experienced at least one episode of inter-regional mobility, and in total 633,825 person-observation years.

### Variables

The dependent variable is a time-varying measure of the likelihood of being employed (as opposed to unemployed or inactive) in any given year *t.* As such, the respondent is considered to be employed (1 = employed; 0 = not employed) in year *t* if they experience an employment episode that overlaps with that year.

The principal independent variable is inter-regional mobility, defined as a move between two different regions within the same country, identified according to the Nomenclature of Territorial Units for Statistics (NUTS 2) classification by Eurostat.^1^ Histories of inter-regional mobility were created using data on the year of migration reported, namely, a change in NUTS 2 region of residence between two consecutive years, resulting in a time-varying variable by year. At the first observation, at 15 years of age, individuals were considered non-movers (0), and upon moving to another region within the same country they were then classified as inter-regional *movers* (1). Individuals who never made an inter-regional move (*stayers*) are grouped with the observations of individuals 2 or more years before the move, always as 0, as they are assumed to have similar circumstances to individuals before they imminently undertake a move. We include only the first inter-regional move for theoretical and empirical motivations: repeat migration is a heavily selected process (Bernard, 2022; Chiswick, 1999), and focusing on the first move allows us to capture the initial effect of geographical mobility on employment outcomes.

To account for family-related commitments when assessing the potential occupational penalty (see Cooke, 2001), we include two time-varying variables capturing partnership and parenthood statuses in a given year. The first variable distinguishes between being single or living with a partner (married or cohabitating)^2^ in the same residence (0 = single, 1 = partnered). This variable is treated as time-varying until the year of mobility, after which it is fixed at the value observed in that year and kept constant for all subsequent years. This approach allows us to focus on the effect of partnership status on individual employment precisely at the time of the first mobility event.^3^ Parenthood status is time-varying, coded as 1 if the individual has one or more children and 0 otherwise, thereby reflecting parenthood as an ongoing status rather than a discrete childbirth event.^4^ However, for most of the analyses, rather than including these two variables separately, we use their interaction, referred to as *family status*, which distinguishes three categories: partnered with children, partnered without children, and single (regardless of parental status).

Additionally, time-varying variables of age as a dummy variable of single years and period, grouped into five categories (1946–1959, 1960s, 1970s, 1980s, 1990–2009), are included in the models to enhance the robustness of results.

### Country Selection

We examine six European countries: France, Germany, Italy, Poland, Spain, and Sweden. The country selection is guided by both data quality and theoretical relevance. Specifically, these countries offer comparable populations and NUTS 2 regional sizes, which makes cross-country comparisons of internal mobility patterns more meaningful. Further, these cases cover the major socio-economic models in European welfare and family systems identified in comparative research: Sweden (Social-democratic welfare regime), Germany and France (Conservative-corporatist welfare regime), Italy and Spain (Mediterranean welfare regime) and Poland (Eastern Europe or (post-)socialist). These countries differ along several institutional and cultural dimensions that are relevant for understanding employment outcomes of geographical mobility, including welfare state regime (Esping-Andersen, 1990), social inequality (Breen & Müller, 2020), support for female employment (Orloff, 1993; Stier et al., 2001), and prominent gender roles and ideology (Pfau-Effinger, 1998, 2012).

As shown in Table 1, geographical mobility in the sample was the most prevalent in Sweden, where, considering men and women together, 42.0% had experienced at least one episode of geographical mobility, and the lowest in Spain (20.6%), Poland (19.1%), and Italy (15.7%). The percentage of movers was intermediate in continental European countries at 33.2% in France and 26.3% in Germany. Countries with higher overall mobility rates also tend to have a higher percentage of single movers. For example, Sweden exhibits both the highest share of movers overall and the highest share of single movers. Across all countries, the lower percentage of female single movers suggests that women are more frequently partnered before or during the year of move compared to men, and that women’s mobility is more likely to coincide with partnership formation or follows a partner’s relocation.

**Table 1:** Percentages of movers, single at move, and sample size by country, separately for men and women

Country	% Movers (all)	% Single movers	*N*
Male			
France	33.8	62.7	1,522
Germany	26.7	66.1	2,004
Italy	16.2	67.7	2,193
Spain	21.1	65.4	2,028
Sweden	38.6	71.2	1,520
Poland	20.8	66.2	2,282
Total	25.0	66.7	11,549
Female			
France	32.8	50.1	2,073
Germany	25.9	50.3	2,213
Italy	15.3	31.8	2,634
Spain	20.2	44.0	2,446
Sweden	44.9	63.3	1,711
Poland	17.6	41.2	2,727
Total	24.6	48.8	13,804

### Methods

We estimated a set of linear probability panel models (LPMs) with fixed effects (FE), which control for unobserved heterogeneity and focus on intra-individual changes over time (Ludwig & Brüderl, 2021). By focusing solely on within-person variation, fixed effects models allow for unbiased estimates despite the existence of unobservable characteristics (e.g., skills, motivation, personality) that are present in processes of (self-)selection, that ultimately influence both geographical mobility and employment status.

The basic model can be formalised as follows:

$$\left( {Y_{{it}} - \bar{Y}_{i} } \right) = \beta \left( {X_{{jit}} - \bar{X}_{{ji}} } \right) + \beta \left( {M_{{it}} - \bar{M}_{i} } \right) + \left( {\alpha _{i} - \bar{a}_{i} } \right) + \left( {\varepsilon _{{it}} - \bar{\varepsilon }_{i} } \right)$$

where the observed changes in employment $\left({Y}_{it}-{\stackrel{-}{Y}}_{i}\right)$ depend on changes in covariates $({X}_{jit}-{\stackrel{-}{X}}_{ji})$ and in inter-regional geographical mobility ($\:{M}_{it}-\stackrel{-}{M}$). Hence, the model focuses on intra-individual changes over time, estimating the effect of geographical mobility on achievement *within* individuals’ life courses and considering only time-varying variables. The key assumption of this model is that the unobserved covariates are time-constant. Models are estimated separately for men and women.

The empirical strategy consists of four models to understand the role of gender, family status, migration timing, and country in shaping the association between geographical mobility and employment status. The first tests *Hypothesis 1* by examining gender differences in returns to mobility in a pooled dataset of all countries, using geographical mobility as a binary variable.

The second model considers the role of family. The objective is to see if the likelihood of being employed is influenced by the intersections between partnership, family status, and geographical mobility (*Hypothesis 2*). Therefore, we add an interaction term between geographical mobility and family status.

The third model considers timing, to explore how the association between geographical mobility and employment is ‘distributed’ over the life course (*Hypotheses 3a* and *3b*), including an interaction between geographical mobility and family status. Geographical mobility is defined as a variable capturing time relative to an individual’s first inter-regional move: two or more years prior to the move (grouped with stayers), one year before, the year of the move, one year after, two to three years after, four to five years after, and six or more years post-move, ensuring sufficient observations at each lead and lag for stable estimation in a finite distributed lag structure, allowing to separately estimate anticipatory effects (pre-move) and adjustment effects (post-move).

The fourth and final model focuses on cross-national differences by estimating the distributed effects of the interaction between mobility and family status over time separately for each country (*Hypotheses 4a* and *4b*).

As robustness checks, we replicated all fixed effects models using random effects specifications, including additional time-constant controls (country, level of education, and social class of parents) and binomial panel logit models with fixed effects. Results confirm the same overall patterns as the main analyses, with only minimal variations in coefficients and significance levels. Results are available upon request.

## Empirical Evidence

### Gender Differences and the Role of Family Status

Table 2 presents results from Model 1, showing the average within-individual change in the probability of being employed after the first move.

**Table 2:** Model 1: Association between binary variable of Inter-regional geographical mobility on employment probability of men and women

	Men		Women	
	Coef	(S.E.)	Coef	(S.E.)
*Geographical mobility*	0.15***	(0.00)	0.09***	(0.00)
[ref: stayer & pre-move]				
*Married/cohabiting*	0.02***	(0.00)	−0.04***	(0.00)
[ref: single]				
*Children*	0.00*	(0.00)	−0.18***	(0.00)
[ref: none]				
Constant	0.32***	(0.00)	0.31***	(0.00)
Observations	288,725		345,100	
R-squared	0.34		0.12	
Number of id	11,549		13,804	

Coefficients with standard errors from linear probability panel models with fixed effects. *Source*: SHARELIFE, authors’ own calculations

***p < 0.01, **p < 0.05, *p < 0.1. Dummy variables for age and period not shown

Estimates suggest a positive and statistically significant association between geographical mobility and employment for both men and women. Moving to another region is associated with a 15-percentage point increase in the probability of being employed for men (Coef.: 0.15, CI: 0.14–0.15) and a 9-percentage point increase for women (Coef.: 0.09, CI: 0.08–0.09). This confirms *Hypothesis 1*, according to which geographical mobility favours men’s employment chances more than those of women. For men, the association between being partnered and employed is marginally positive (Coef.: 0.02, CI: 0.02–0.02) but the association with having children is not statistically significant. In contrast, for women, being partnered is associated with a 4-percentage point lower probability for employment compared to single women (Coef.: −0.04, CI: −0.05– −0.04). Such gendered familial aspects are further highlighted by the parenthood status coefficient, which is even more strongly negative (Coef.: −0.18, CI: −0.18– −0.17). Overall, while geographical mobility is associated with higher employment among men and single women, confirming Hypothesis 1, women’s employment appears to be more strongly conditioned by family circumstances than men’s.

To obtain a more granular understanding of the interplay between geographical mobility and family status, we incorporate an interaction term between geographical mobility and family status (single, partnership without children, partnership with children) in Model 2. Table 3 reports the results. While childless partnership is associated with increased employment opportunities for both men (Coef.: 0.05, CI: 0.05–0.06) and women (Coef.: 0.03, CI: 0.02–0.03), the role of parenthood is strongly gender-differentiated: mothers experience a 16-percentage-point reduction in employment (Coef.: −0.16, CI: −0.16– −0.15), whereas fatherhood is associated with a 2-percentage point increase in employment likelihood (0.02, CI: 0.02–0.03). However, once geographical mobility is considered, the gendered outcomes of parenthood are no longer evident: both partnered men and partnered women experience reductions in employment chances, regardless of whether or not they have children. Considering the interaction term, fathers and childless partnered men show similarly reduced coefficients (Coef.: −0.10, CI: −0.11– −0.08; −0.11, CI: −0.12– −0.10), similar to mothers and childless partnered women (Coef.: −0.12, CI: −0.14– −0.11), suggesting that partnership status, rather than parenthood, drives this effect.

**Table 3:** Model 2: Association between binary variable of Inter-regional geographical mobility on employment probability of men and women with interaction between mobility and family status

	Men		Women	
	Coef	(S.E.)	Coef	(S.E.)
*Geographical mobility*	0.19***	(0.00)	0.15***	(0.00)
[ref: stayer & pre-move]				
*Family status* [ref: single]				
Partnership, no children	0.05***	(0.00)	0.03***	(0.00)
Partnership & children	0.02***	(0.00)	−0.16***	(0.00)
Interaction GeoMob * Family status				
Move * Partnership, no children	−0.10***	(0.01)	−0.12***	(0.01)
Move * Partnership & children	−0.11***	(0.01)	−0.12***	(0.01)
Constant	0.32***	(0.00)	0.31***	(0.00)
Observations	288,725		345,100	
R-squared	0.35		0.12	
Number of id	11,549		13,804	

Coefficients with standard errors from linear probability panel models with fixed effects. *Source*: SHARELIFE, authors’ own calculations

***p < 0.01, **p < 0.05, *p < 0.1. Dummy variables for age and period not shown. Results separately by country are available in Table 4 in the Appendix

This indicates that mobility interacts with family status in ways that offset the advantages of partnership. In other words, while partnership is generally associated with better employment outcomes, this advantage disappears when coupled with mobility. This suggests that moving imposes constraints or disruptions that particularly affect partnered women, regardless of whether or not they have children. This finding, which supports Hypothesis 2, indicates that bargaining constraints within partnerships limit women’s employment after a move, whereas single women have more flexibility and thus higher chances of being employed.

### The Association Between Geographical Mobility and Employment Over Time

**Fig. 1:**
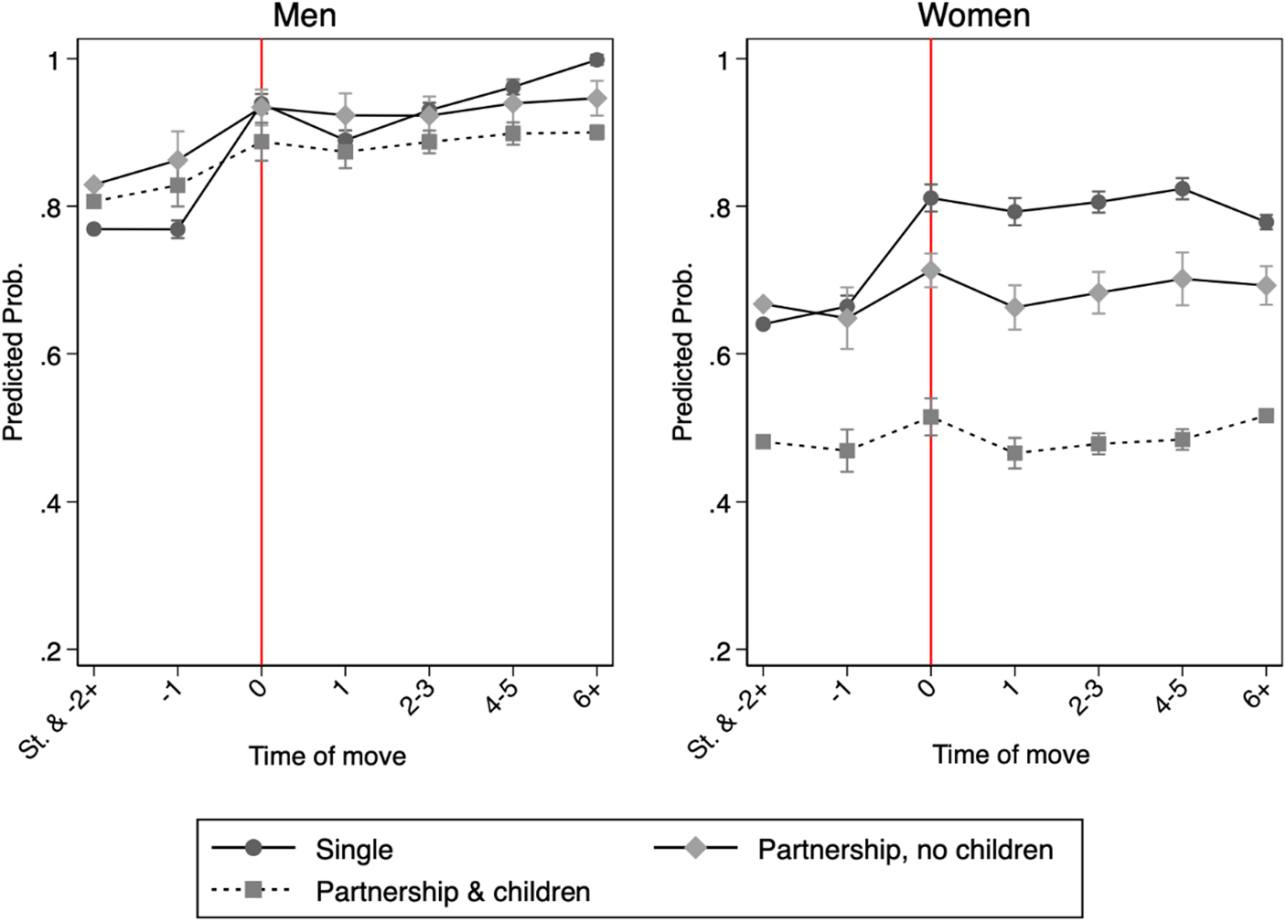
Model 3: Predicted probabilities of employment by gender and family status with 95% confidence intervals. Results from distributed fixed effects models with an interaction between geographical mobility and family status. Predictions are computed at each individual’s observed covariate values and averaged across the sample

Proceeding with the third model, we instead look at the association between geographical mobility and employment over time, separately for men and women (Fig. 1). The x-axis measures years before and after a move. The y-axis shows the predicted employment status computed at each individual’s observed covariate values and averaged across the sample. The three lines correspond to different combinations of partnership and parental status.

Men report similar employment trajectories regardless of their parental status. Single men start at the lowest predicted probabilities pre-move, but gain the most at the year of the move and beyond, reaching the highest predicted probabilities six or more years post-move. Partnered men also experience gains in the year of the move, but the increase is less pronounced and remains steady over time. Childless partnered men maintain consistently higher levels of predicted employment, often overlapping with those of single men, while fathers show the lowest levels of employment post-move, but rather high levels overall. Overall, geographical mobility over time benefits men’s employment, with rather minor variations by family status, validating Hypothesis 3b.

Among women, pre-move employment levels of single and childless partnered women are similar, while mothers are substantially less likely to be employed. At the year of migration, employment increases sharply for single women and remains high afterwards. In contrast, partnered women, regardless of parental status, experience only a temporary employment improvement in the year of migration, with post-move employment levels returning close to their pre-move values. These patterns suggest that single women benefit most from mobility, likely reflecting their employment-related motivations for moving, whereas partnered women may move primarily for family reasons, experiencing limited changes in employment chances. Overall, results do not support a pattern of continued disadvantage for partnered women as predicted by *Hypothesis 3a*.

### Country-Specific Variations

**Fig. 2:**
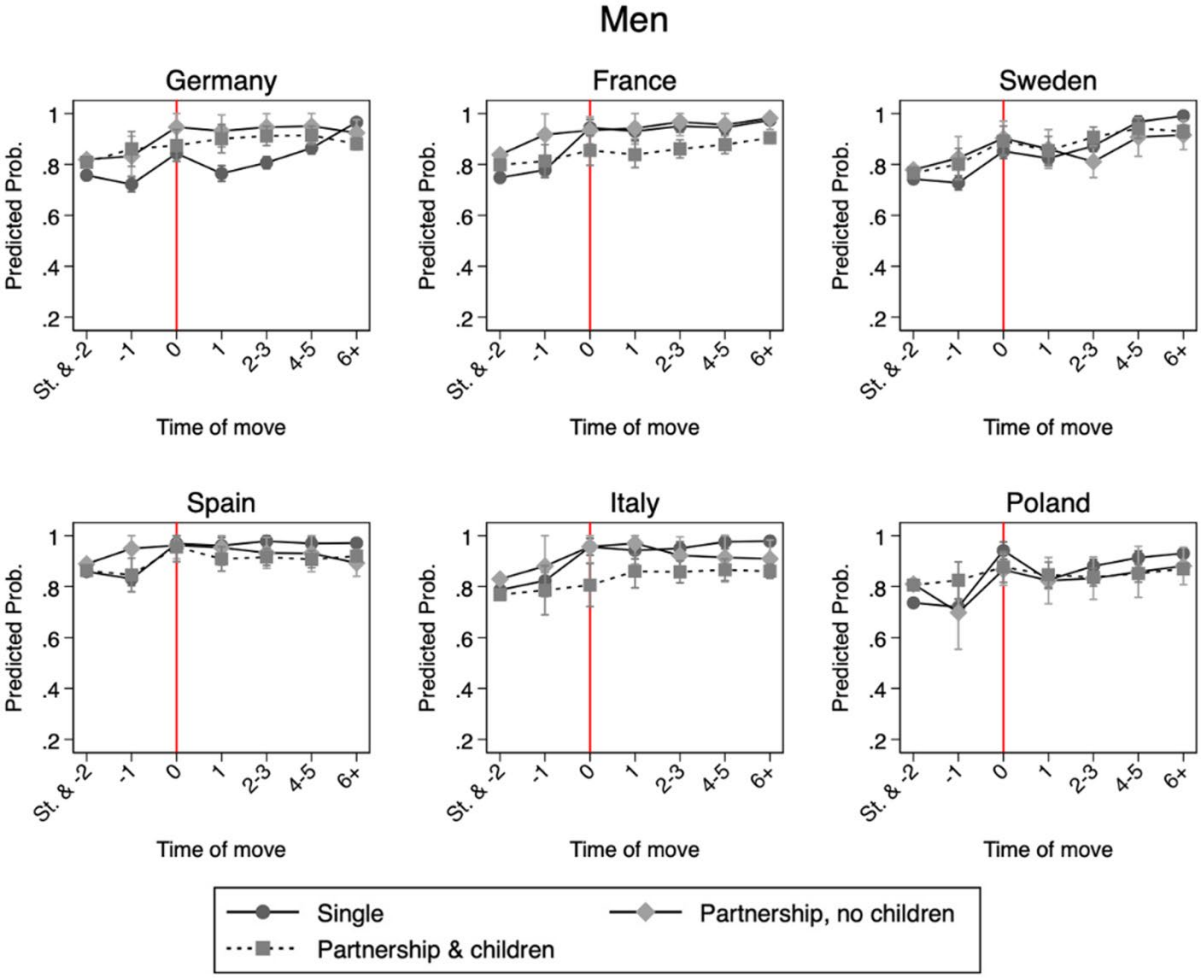
Model 4: Predicted probabilities of employment for men by country, with 95% confidence intervals. Results from distributed fixed effects models with an interaction between inter-regional geographical mobility and partnership status. Red line indicating the year of first inter-regional move. Predictions are computed at each individual’s observed covariate values and averaged across the sample

For the fourth model, Figs. 2 (men) and 3 (women) show predicted probabilities of employment in a cross-national perspective. For men, family status is associated with a limited change in employment probability post-move, and the differences between childless men and fathers are largely not statistically significant. Overall, among men, regardless of their family status, employment patterns over time are consistent across national contexts, confirming Hypothesis 4b.

In contrast, among women, the outcomes of geographical mobility vary by family status. In most countries, single women consistently show the highest employment levels compared to partnered women and experience an increase in predicted employment in the year of migration. Among mothers, the employment probabilities are the lowest, with little or no change post-move. Exceptions are Germany, where childless partnered women have the highest predicted employment levels during the first few years post-migration, and Poland, where single and childless partnered women experience similar employment trajectories, while mothers show substantially lower employment probabilities.

Regarding Hypothesis 4a, Spain and Italy exhibit the largest employment penalties for partnered women and mothers compared to single women. This reflects that mobility increases employment for single women while reducing it for partnered women, whereas mothers remain at consistently low employment levels regardless of mobility status. Conservative-corporatist countries show a similar pattern, with mothers facing a stronger mobility-related penalty, while childless partnered women are more likely to be employed. By contrast, differences in family status are smaller in Sweden and Poland, where partnered and single women have similar post-move employment probabilities and mothers are the only group with systematically lower chances of employment. Overall, Hypothesis 4a is only partially confirmed: employment penalties for partnered women and mothers are lowest in Sweden and highest in Mediterranean countries, but France and Germany deviate less from this pattern than expected, while Poland trends more closely to Sweden than to other countries.

**Fig. 3:**
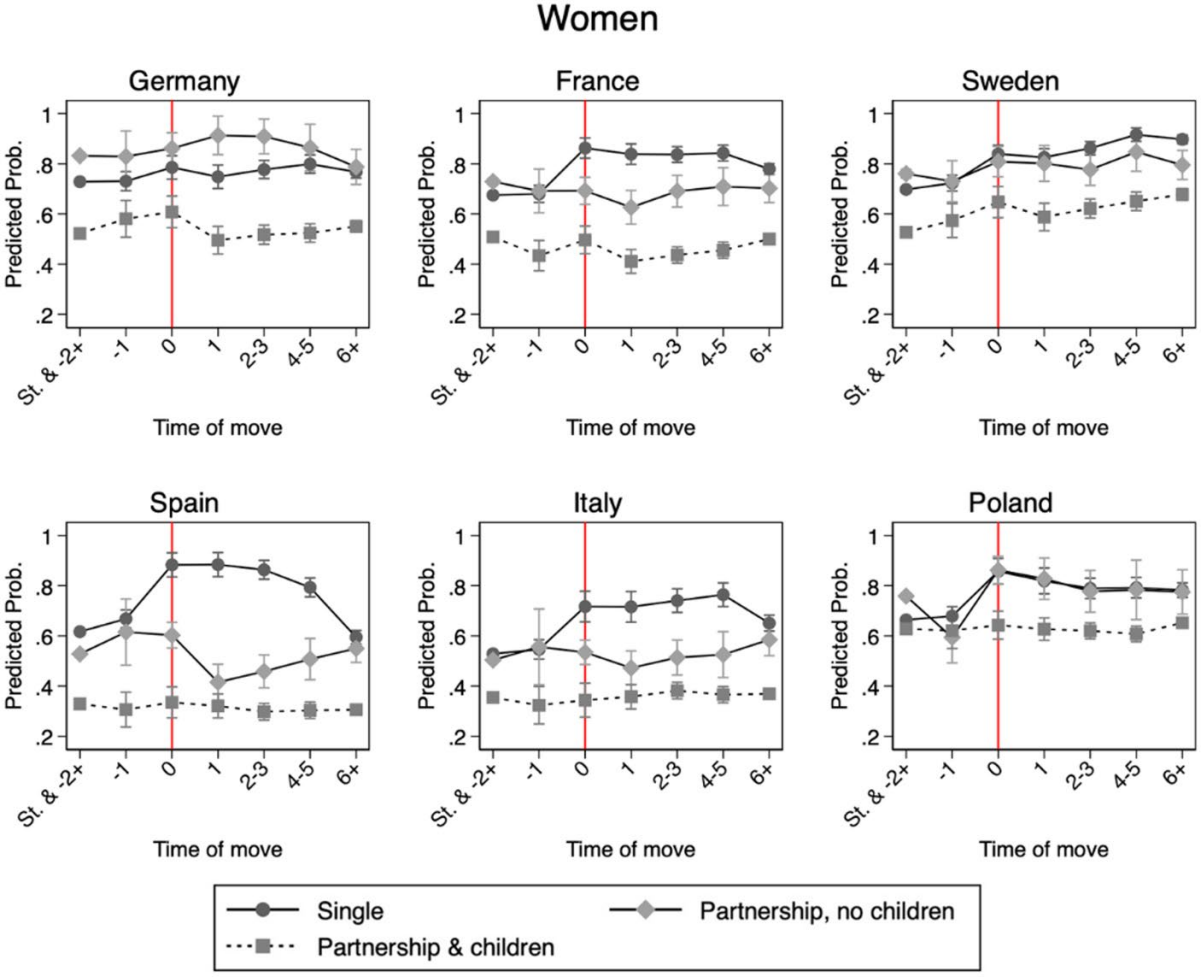
Model 4: Predicted probabilities of employment for women by country, with 95% confidence intervals. Results from distributed fixed effects models with an interaction between inter-regional geographical mobility and family status. Red line indicating the year of first inter-regional move. Predictions are computed at each individual’s observed covariate values and averaged across the sample

## Discussion and Conclusion

This study offers a comparative analysis of the role of gender and family status in shaping the association between inter-regional mobility and employment status over time in France, Germany, Italy, Poland, Spain, and Sweden. Findings support *Hypothesis 1*, indicating that the positive association between geographical mobility and employment is more evident for men than for women. Geographical mobility can improve women’s employment probabilities; however, this improvement is contingent on individual and contextual factors that are assessed in the subsequent hypotheses.

In line with the literature on gender and family differences in geographical mobility, the study shows that partnered women, both with and without children, are overall less likely to be employed than single women following geographical mobility (*Hypothesis 2* confirmed). This aligns with the expectation that women with family obligations face less favourable employment outcomes due to gendered dynamics of decision-making, family commitments, and the prioritisation of male partners' labour market opportunities within couples. At times, partnership is associated with better employment outcomes, but this benefit seems to disappear once geographical mobility is introduced. Moving appears to impose constraints or disruptions that affect primarily partnered women, suggesting that bargaining processes within families may limit flexibility in finding employment post-move, while single men are better able to adapt to new circumstances.

According to *Hypothesis 3a*, it was expected that mothers would experience particularly negative employment outcomes in the long-term due to the combined influence of motherhood and couple migration accumulating over time. Results, however, do not provide clear support for this hypothesis. Mothers consistently display lower employment levels than childless partnered and single women, but mobility itself does not further reduce their prospects over time. The only group benefiting from mobility in a sustained way are women who were single at the time of the move, suggesting stronger employment-related motivations for moving. By contrast, partnered women, regardless of their parental status, show little change in employment after moving. Overall, geographical mobility does not further reduce employment chances of mothers, but it does reinforce existing inequalities among women by advantaging single women. In contrast, men, regardless of their family status, experience a gradual increase in employment opportunities over time following migration (*Hypothesis 3b* confirmed).

One of the main strengths of this analysis lies in its comparative perspective. Examining each country individually, we find that among men, regardless of their family status, employment trajectories following migration are consistent across national contexts (*Hypothesis 4b* confirmed). Conversely, among women, the employment consequences of geographical mobility vary substantially across welfare state regimes. While the strongest penalties for partnered women and mothers clearly emerge in Mediterranean countries and the weakest emerge in Sweden, patterns in Central European countries diverge less from the Mediterranean model than anticipated. Specifically, the trajectory observed in Poland aligns more closely with the Swedish case, suggesting that post-socialist contexts do not entirely fit traditional welfare state regime classifications. Taken together, these findings provide only partial support for *Hypothesis 4a* and highlight the need to move beyond broad welfare typologies to capture the nuanced ways in which institutional contexts shape the gendered outcomes of mobility. Nevertheless, this study offers an important contribution by providing a cross-national examination of the intricate relationship between geographical mobility, employment, and family status from a gender-sensitive perspective across diverse national contexts.

An important consideration to the study is that due to the use of SHARELIFE data, which collects retrospective information on individuals born up to the 1960s, our results do not capture recent or contemporary dynamics. Instead, they reflect the experiences of cohorts who were active in the labour market primarily during the second half of the twentieth century, a period in which women’s outcomes were more strongly shaped by structural inequalities and institutional barriers. More recent birth cohorts may show different patterns due to declines in marriage and fertility rates, shifts in gender roles, changes in family-support policies, and evolving labour market structures. For example, greater female labour force participation and more flexible work arrangements may reduce the employment gap between mothers and childless women post-mobility, while persistent gender norms in some countries may continue to disadvantage mothers. When more recent comparative data become available, further analyses will be needed to assess whether results remain the same.

An additional consideration is that the study focuses only on the first inter-regional move after age 15, even though geographical mobility can be a much more complex process involving moves to other locations (Mulder & van Ham, 2005). After the first move, individuals do not necessarily remain in the new location; some may choose to return to their places of origin or move on to other places. Additionally, our study cannot distinguish moves motivated by partner’s employment from those driven by the individual’s own career, which may limit the ability to isolate gendered effects, so future research should focus on these mechanisms.

## Data Availability

This paper uses data from SHARE Waves 3 and 7. The SHARE data collection has been funded by the European Commission, DG RTD through FP5 (QLK6-CT-2001-00360), FP6 (SHARE-I3: RII-CT-2006-062193, COMPARE: CIT5-CT-2005-028857, SHARELIFE: CIT4-CT-2006-028812), FP7 (SHARE-PREP: GA N°211909, SHARE-LEAP: GA N°227822, SHARE M4: GA N°261982, DASISH: GA N°283646) and Horizon 2020 (SHARE-DEV3: GA N°676536, SHARE-COHESION: GA N°870628, SERISS: GA N°654221, SSHOC: GA N°823782, SHARE-COVID19: GA N°101015924) and by DG Employment, Social Affairs & Inclusion through VS 2015/0195, VS 2016/0135, VS 2018/0285, VS 2019/0332, VS 2020/0313, SHARE-EUCOV: GA N°101052589 and EUCOVII: GA N°101102412. Additional funding from the German Federal Ministry of Education and Research (01UW1301, 01UW1801, 01UW2202), the Max Planck Society for the Advancement of Science, the U.S. National Institute on Aging (U01_AG09740-13S2, P01_AG005842, P01_AG08291, P30_AG12815, R21_AG025169, Y1-AG-4553-01, IAG_BSR06-11, OGHA_04–064, BSR12-04, R01_AG052527-02, R01_AG056329-02, R01_AG063944, HHSN271201300071C, RAG052527A) and from various national funding sources is gratefully acknowledged (see www.share-eric.eu).

## Appendix

See Table 4.

**Table 4:** Association between binary variable of inter-regional geographical mobility on employment probability of men and women with interaction between mobility and family status, by country

	Germany					Sweden							Spain						
	Men		Women			Men			Women				Men				Women		
	Coef	(S.E.)	Coef		(S.E.)	Coef.		(S.E.)	Coef.		(S.E.)		Coef.		(S.E.)		Coef.		(S.E.)
*Geographical mobility*	0.14***	(0.01)	0.04***		(0.01)	0.21***		(0.01)	0.17***		(0.01)		0.15***		(0.01)		0.09***		(0.01)
[ref: stayer & pre-move]																			
*Family status* [ref: single]																			
Partnership, no children	0.05***	(0.01)	0.10***		(0.01)	0.01*		(0.01)	0.06***		(0.01)		0.03***		(0.01)		−0.07***		(0.01)
Partnership & children	0.03***	(0.01)	−0.21***		(0.01)	−0.02**		(0.01)	−0.18***		(0.01)		0.00		(0.00)		−0.25***		(0.01)
*Interaction GeoMob * Family status*																			
Move * Partnership, no children	−0.03	(0.02)	−0.01		(0.02)	−0.13***		(0.02)	−0.14***		(0.02)		−0.10***		(0.02)		−0.09***		(0.02)
Move * Partnership & children	−0.06***	(0.01)	−0.03*		(0.02)	−0.07***		(0.01)	−0.05***		(0.01)		−0.09***		(0.01)		−0.11***		(0.02)
Constant	0.20***	(0.01)	0.34***		(0.01)	0.36***		(0.01)	0.35***		(0.01)		0.60***		(0.01)		0.44***		(0.01)
Observations	50,100		55,325			38,000			42,775				50,700				61,150		
R-squared	0.41		0.15			0.34			0.21				0.19				0.07		
Number of id	2,004		2,213			1,520			1,711				2,028				2,446		

	Italy					France								Poland						
	Men		Women			Men			Women					Men				Women		
	Coef	(S.E.)	Coef		(S.E.)	Coef.		(S.E.)	Coef.		(S.E.)			Coef.		(S.E.)		Coef.		(S.E.)
*Geographical mobility*	0.22***	(0.01)	0.15***		(0.02)	0.20***		(0.01)	0.14***		(0.01)			0.18***		(0.01)		0.12***		(0.01)
[ref: stayer & pre-move]																				
*Family status* [ref: single]																				
Partnership, no children	0.05***	(0.01)	−0.02***		(0.01)	0.09***		(0.01)	0.06***		(0.01)			0.07***		(0.01)		0.09***		(0.01)
Partnership & children	−0.01**	(0.00)	−0.17***		(0.01)	0.04***		(0.01)	−0.16***		(0.01)			0.06***		(0.00)		−0.03***		(0.01)
*Interaction GeoMob * Family status*																				
Move * Partnership, no children	−0.09***	(0.02)	−0.13***		(0.02)	−0.08***		(0.02)	−0.18***		(0.02)			−0.13***		(0.02)		−0.05**		(0.03)
Move * Partnership & children	−0.13***	(0.02)	−0.14***		(0.02)	−0.11***		(0.01)	−0.16***		(0.01)			−0.12***		(0.01)		−0.11***		(0.02)
Constant	0.36***	(0.01)	0.24***		(0.01)	0.25***		(0.01)	0.27***		(0.01)			0.15***		(0.01)		0.16***		(0.01)
Observations	54,825		65,850			38,050			51,825					57,050				68,175		
R-squared	0.35		0.08			0.40			0.15					0.43				0.29		
Number of id	2,193		2,634			1,522			2,073					2,282				2,727		

Coefficients with standard errors from linear probability panel models with fixed effects. *Source*: SHARELIFE, authors’ own calculations

***p < 0.01, **p < 0.05, *p < 0.1. Dummy variables for age and period not shown

## Footnotes

1 We do not consider intra-regional moves, as these typically involve shorter distances that are less likely to alter individual’s employment situations, allowing for commuting from home to work. We also do not look at migration between countries, as individuals then face a new set of circumstances and motivations which are not accounted for in this study.

2 We include partners who were cohabiting in the same residence alongside marriage in order to account for potential country-level differences, in which marriage was more frequent in certain countries and cohabitation favored in others. Levels of cohabitation are low at about 3% of unmarried individuals overall.

3 Partnership for stayers can vary throughout all years of observation, as there is no mobility event in the life course.

4 Children are treated as a time-varying variable throughout the observation period, since fixing this characteristic at the time of migration would not have allowed us to capture the birth of a child after migration—an event that can be highly relevant in shaping the likelihood of being employed.
